# Case Report: A case series of Lhermitte–Duclos disease with surgical intervention

**DOI:** 10.3389/fonc.2025.1552495

**Published:** 2025-10-23

**Authors:** Ziyang Chen, Min Guo, Xingke Li, Cheng Lin, Guili Feng, Jianzhi Zhou, Hainan Li, Baijie Chen, Lirong Liu, Linbo Cai, Lei Wang, Hui Ouyang, Yanfeng Fan

**Affiliations:** ^1^ Department of Neurosurgery, Guangdong Sanjiu Brain Hospital, Guangzhou, China; ^2^ Institute of Clinical Teaching Center, Jinan University, Guangzhou, China; ^3^ Institute for Brain Research and Rehabilitation, South China Normal University, Guangzhou, China; ^4^ Department of Pathology, Guangdong Sanjiu Brain Hospital, Guangzhou, China; ^5^ Department of Rehabilitation, Guangdong Sanjiu Brain Hospital, Guangzhou, China; ^6^ Department of Oncology, Guangdong Sanjiu Brain Hospital, Guangzhou, China

**Keywords:** Lhermitte–Duclos disease, dysplastic cerebellar gangliocytoma, Cowden syndrome, hamartoma, surgical intervention, case report

## Abstract

**Background:**

Lhermitte–Duclos disease (LDD) is a rare dysplastic cerebellar gangliocytoma often associated with Cowden syndrome and phosphatase and tensin homolog (PTEN) alterations. We report a three-case series focusing on imaging, histopathology, PTEN testing, surgical decision-making, and outcomes.

**Method:**

We retrospectively identified three adults with LDD who underwent standardized preoperative imaging [including magnetic resonance spectroscopy (MRS) and perfusion when feasible], surgery, and structured follow-up with Karnofsky Performance Status (KPS). PTEN assessment included immunohistochemistry and/or genetic testing where available.

**Results:**

All patients in this case series were women (18–53 years). Two underwent subtotal resection and one underwent gross total resection. Characteristic “tiger-striped” magnetic resonance imaging (MRI) appearance was present in all cases. Histopathology showed thickened molecular layer, loss of Purkinje cells, and hypertrophic ganglion-like neurons. One patient required unplanned posterior fossa decompression due to severe postoperative edema. At 6 months, two patients improved functionally while one had poor neurological outcome. PTEN testing supported the association with PTEN hamartoma tumor syndrome in one case.

**Conclusion:**

PTEN evaluation should be considered in adults with LDD, especially when clinical features suggest Cowden syndrome. Surgical management should balance extent of resection with preservation of venous outflow and cerebellar function. Non-surgical strategies [observation, stereotactic radiotherapy, and exploratory mechanistic target of rapamycin (mTOR) inhibition] may be an option in selected scenarios.

**Limitations:**

This single-center retrospective series is limited by its small sample size and variable follow-up imaging.

## Introduction

### Definition and clinical relevance

Lhermitte–Duclos disease (LDD) is an infrequently diagnosed benign tumor of the posterior cranial fossa (1). In particular, it is accompanied by increased morbidity and mortality in those aged less than 40 years with no apparent predominance across genders (2–4). A wide range of neurological symptoms and cerebellar signs are present in LDD (5, 6). To date, the perioperative neurological function remains poor for patients with LDD due to its insidious onset in the infratentorial region (7). Surgical resection is usually chosen in the management of symptomatic patients with LDD, especially those who develop signs and symptoms such as increased intracranial pressure and progressive mass effect (8). In the case of complete resection without new-onset complications, the overall prognosis is encouraging (9). The key histopathological characteristic of LDD is the replacement of the granular cell layer with dysplastic cortical neurons, which drives diffuse hypertrophy of the cerebellar cortex, with a distinctive layered pattern called “Tiger-striped appearance” (1, 10).

Emerging evidence has shown that those suffering from LDD have a significant correlation with Cowden syndrome (CS) (11). A particularly aggressive subtype of multiple hamartoma syndrome, also known as phosphatase and tensin homolog (PTEN) hamartoma tumor disorder (12), leads to hereditary cancer predisposition and overgrowth disorder, which subsequently drives multisystem hamartomas and, eventually, malignancies (9). Therefore, patients with CS are at increased risk for breast, thyroid, digestive system, genitourinary, and endometrial carcinoma (13) and multi-hamartomatous lesions (14). The pathogenesis and epidemiological features of LDD still require further investigation.

### Outcome measures and follow-up schedule

This retrospective case series was conducted at Guangdong Sanjiu Brain Hospital from June 2021 to December 2023 (**Supplemetary Table 1**). We systematically reviewed our institutional database to identify patients with LDD who underwent surgical intervention. The diagnosis was primarily based on characteristic magnetic resonance imaging (MRI) findings and subsequently confirmed by histopathological examination following surgical resection. For inclusion in this study, patients were required to have complete medical records encompassing preoperative evaluation, surgical details, and postoperative follow-up data, with a minimum follow-up period of 6 months. We excluded cases with incomplete medical records or imaging data, those without histopathological confirmation, patients with follow-up periods shorter than 6 months (follow-up outcomes: neurological status at 1, 3, and 6 months, imaging findings, and quality of life assessment), and individuals who had previously undergone surgical intervention for LDD at other institutions.

All patients included in the study underwent a standardized preoperative evaluation protocol. This comprehensive assessment included detailed neurological examination, contrast-enhanced MRI studies, and magnetic resonance spectroscopy (MRS) when clinically indicated. MRS was employed preoperatively to confirm the radiological diagnosis and rule out other posterior fossa pathologies. The characteristic metabolic profile helped differentiate LDD from other cerebellar lesions, particularly important due to some atypical imaging features on conventional MRI. Based on our experience and prior practice patterns, we suggest the following pragmatic surveillance framework: (i) subtotal resection or known residual—obtain a baseline MRI at 3–6 months, then repeat every 6–12 months for at least 3–5 years; (ii) gross total resection—obtain a baseline MRI at 3–6 months, then annually for 3–5 years; (iii) asymptomatic or mildly symptomatic patients managed conservatively—annual MRI, advancing the interval if symptoms evolve; and (iv) coexistent or strongly suspected PTEN/CS—adhere to the above neuroimaging schedule and integrate system−level oncologic surveillance. For each patient, we documented the total follow−up time (months) and the timing and radiologic conclusion of the latest MRI examination.

Genetic testing for PTEN mutation was performed when feasible, considering its known association with LDD (15, 16). The decision for surgical intervention was based on multiple factors and carefully evaluated for each patient. Primary considerations included the presence of neurological symptoms, documented progression in lesion size, evidence of mass effect on surrounding structures, and patient preference following thorough discussion of potential risks and benefits. To objectively evaluate neurological function and treatment outcomes, the Karnofsky Performance Status (KPS) scale was employed. This standardized approach ensured consistent decision-making across all cases while allowing for individualization based on specific patient factors.

## Case series

### Case 1

**Figure 1:**
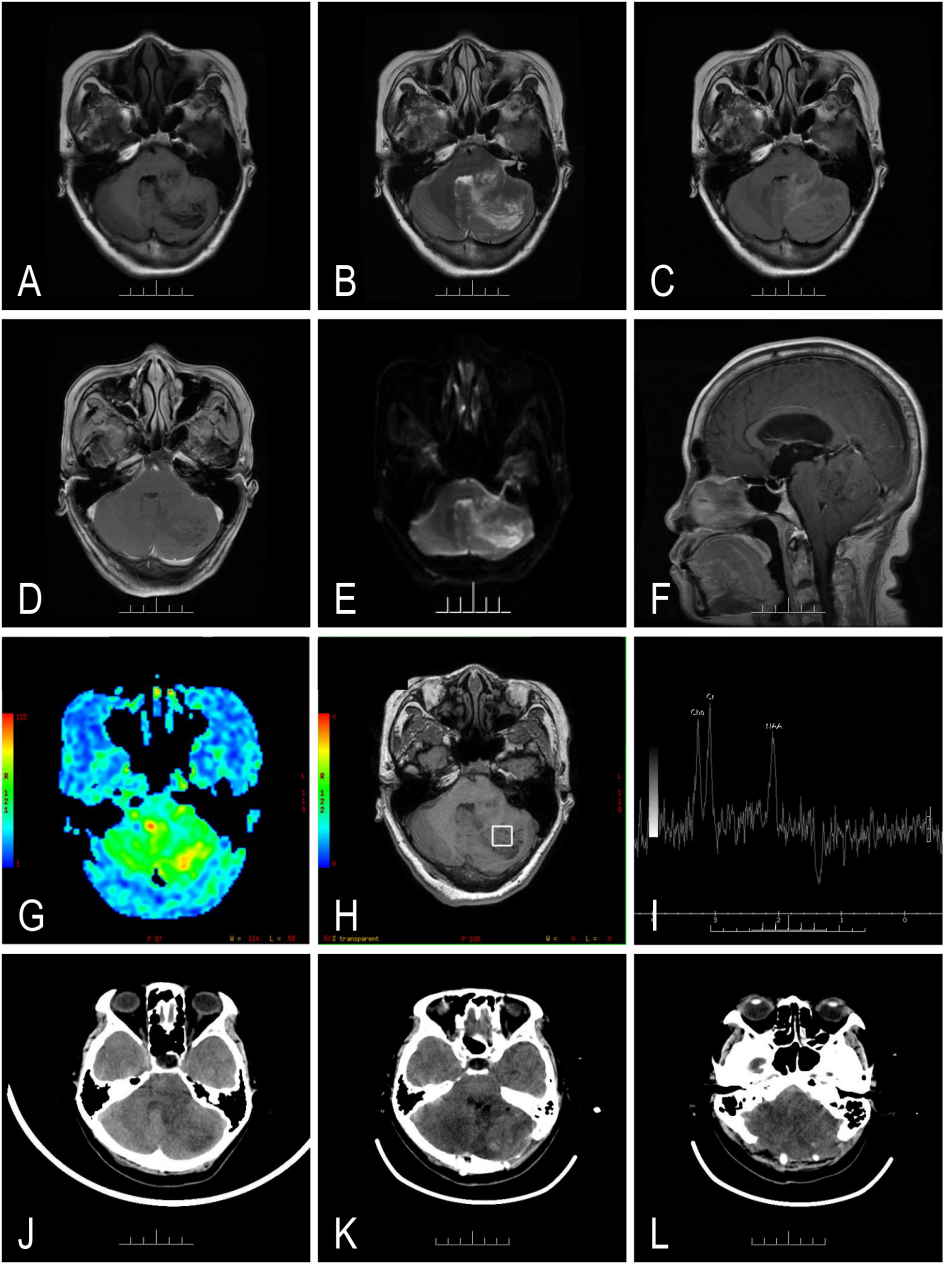
Representative imaging of case 1 in the perioperative period. It showed a heterogeneous lesion with T1 hypointense **(A)** and T2 hyperintense **(B)** abnormal signal shadows in the left cerebellar hemisphere–vermis cerebelli–left pontine arm. The lesion shows slight edema and “tiger-striped appearance” in T2- and fluid-attenuated inversion recovery (FLAIR)-weighted imaging **(C)**, mild linear contrast-enhancement in T1 enhancement **(D)**, and non-diffusion restriction in susceptibility in diffusion-weighted imaging (DWI) **(E)**. The fourth ventricle is displaced and the cerebellar tonsil is sharpened and descended **(F)**. Arterial spin labeling (ASL) presents isoperfusion in the target area **(G)**. Magnetic resonance spectroscopy imaging (MRSI) shows decreased N-acetylaspartate **(H, I)**. The lesion shows non-calcifications or hemorrhage in computed tomography (CT) **(J)**. Unfortunately, severe cerebellar tissue edema occurred after the subtotal resection (STR) **(K)** and then the patient underwent decompressive craniectomy **(L)**.

A 53-year-old woman had recurrent headaches for 3 years. Upon admission, MRI suggested an occupancy lesion in the left cerebellar hemisphere–cerebellar vermis–left pontine arm, considering a heterogeneous lesion with T1 hypointense (**Figure 1A**) and T2 hyperintense signals, also called “tiger-striped sign” (**Figures 1B, C**). Enhancement sequence showed locally suspicious mild linear enhancement (**Figure 1D**), and after DWI scan, an uneven slightly high signal was shown (**Figure 1E**). It was accompanied by supratentorial obstructive hydrocephalus and periventricular interstitial edema (**Figure 1F**). The occupying lesion is mainly shown as patchy pseudocolor green perfusion, interspersed with patchy pseudocolor red high-perfusion areas (**Figure 1G**). Taking the lesion in the left cerebellar hemisphere as the region of interest, NAA peak is decreased (**Figures 1H, I**). Then cranial CT showed non-calcifications or hemorrhage inside occupancy lesions (**Figure 1J**). After excluding surgical contraindications, subtotal excision was performed (**Figure 1K**). The surgical approach involved a left lateral sub-occipital craniotomy with the patient in prone position. Intraoperative neuronavigation was utilized for precise localization. The lesion was approached through the vermian corridor, carefully preserving the surrounding vasculature. The characteristic enlarged folia were identified, and microsurgical dissection was performed along the interface between normal and pathological tissue. Sub-total resection was achieved using a microsurgical technique with continuous neurophysiological monitoring. Postoperatively, the patient developed cerebellar edema, likely attributed to manipulation of deep cerebellar white matter and temporary venous congestion during surgery. While no direct venous sinus injury occurred, the extensive dissection required near the torcula and transverse sinus might have contributed to temporary venous outflow disruption. The edema was managed with high-dose dexamethasone and hyperosmolar therapy. Despite aggressive medical management, the patient experienced persistent ataxia, highlighting the challenging nature of surgery in this eloquent location. Because of significantly increased intracranial edema and enlarged ventricles compared to pre-operation, the patient underwent an unplanned posterior cranial fossa decompression (**Figure 1L**). Preoperatively, the patient presented with a KPS score of 60, requiring occasional assistance with daily activities due to persistent headache. After treatment, at 1-month, 3-month, and 6-month follow-up, the patient remained in a deep coma, with no cough reflex and spontaneous breathing. The total follow-up is 6 months (telephone-based), and no postoperative MRI was obtained.

### Case 2

**Figure 2:**
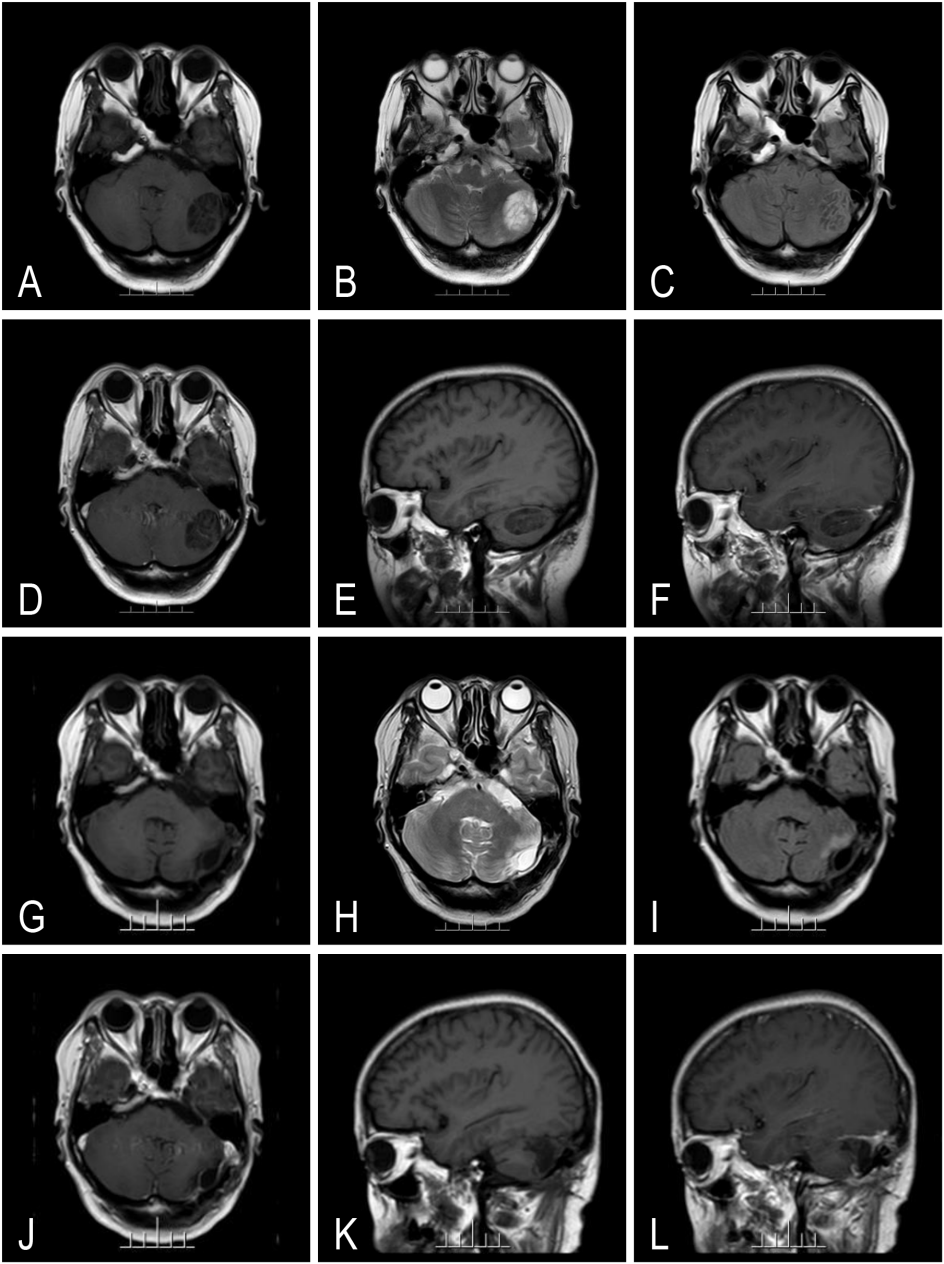
Representative magnetic resonance imaging of case 2 with gross total resection (GTR). It showed a heterogeneous lesion with T1 hypointensity **(A)** and T2 hyperintensity **(B)** in the left cerebellum. The lesion shows non-edema **(C)** and non-contrast enhancement **(D)** but a striated folial pattern (tiger-striped appearance) **(C)** in FLAIR or T1-weighted imaging. The tumor was confined to one side of the cerebellar hemisphere according to sagittal projections **(E, F)** and was completely GTR **(G-L)**.

A 44-year-old female patient started experiencing progressive headaches for 1 month. MRI showed a mass-like abnormal signal in the left cerebellar hemisphere, with uneven T1 hypointense (**Figure 2A**) and T2 hyperintense (**Figure 2B**) abnormal signals, no significant edema was seen around the lesion, a “tiger-striped sign” was seen in the FLAIR sequence (**Figure 2C**), no abnormal enhancement was observed (**Figure 2D**), and no herniation of the cerebellar tonsils was seen in the sagittal plane (**Figures 2E, F**). The decision for surgical intervention was based on several critical factors: First, headaches over months demonstrated the progression of the lesion. Second, the patient’s young age (44 years) and excellent functional status made early intervention preferable to prevent future neurological deterioration. After completing related examinations, the patient underwent a retrosigmoid approach and gross total surgery to remove the space-occupying lesion in the left cerebellar hemisphere (**Figures 2G–L**). After careful dissection of the cerebellar tonsils, we encountered the characteristic widened folia with their distinctive vascular pattern. The lesion was removed through systematic debulking and careful dissection of the margins, with particular attention to preserving the deep cerebellar nuclei and their outflow tracts. Intraoperative ultrasound was used to confirm the extent of resection. No neurological deficits were observed and no systemic complications occurred; overall outcomes were favorable. The patient presented with a preoperative KPS score of 60, requiring considerable assistance due to severe headache. Following gross total resection, her KPS score improved to 70 at 1-month follow-up. By 3 months postoperatively, further improvement to 80 was noted. At 6-month follow-up, her KPS score reached 90, indicating that she could resume normal activities with effort. The total follow-up period was 48 months (telephone-based); the latest postoperative MRI was performed at month 6 (**Figures 2G–L**).

### Case 3

**Figure 3:**
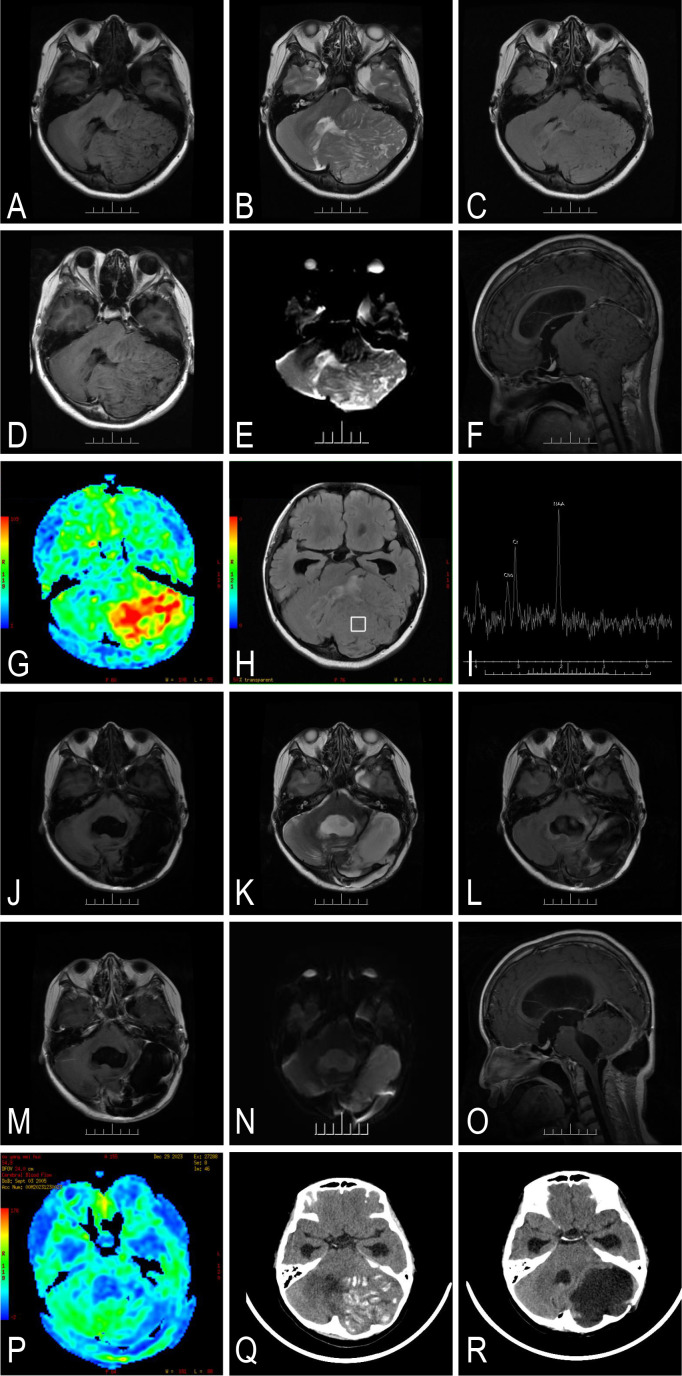
Representative imaging of case 3 in the perioperative period. It showed a heterogeneous lesion with T1 hypointense **(A)** and T2 hyperintense **(B)** abnormal signals in the left cerebellum, brainstem, and spinal canal. A slight edema and “tiger-striped appearance” in T2- and fluid-attenuated inversion recovery (FLAIR)-weighted imaging **(C)**, heterogeneous linear contrast enhancement in T1-weighted imaging **(D)**, and non-diffusion restriction in DWI are shown **(E)**. The fourth ventricle is displaced, the cerebellar tonsil is compressed and deformed, and the tumor grew into the spinal canal at the C2 level **(F)**. ASL imaging presents hyper-transfusion in the target area, suggesting a rich blood supply **(G)**. The results of MRSI exclude glioma and medulloblastoma **(H, I)**. The lesion shows multiple heterogeneous strip-like calcifications in CT **(Q)**. The patient obtained STR, and part of the tumor remains on the side of the brainstem and below the pineal gland for preservation of neurological function **(J–P, R)**.

An 18-year-old woman began to experience paroxysmal progressive dizziness 3 years ago, accompanied by unsteady walking and a gradual decrease in hearing on the left side, along with coughing while drinking water. Physical examination found damage to the left vestibulocochlear nerve, left glossopharyngeal nerve, left hypoglossal nerve, and bilateral vision and visual fields. MR examination showed a huge occupying lesion in the left cerebellar hemisphere–brainstem, extending downward into the spinal canal reaching the level of the C2 vertebra, with secondary foramen magnum herniation and supratentorial obstructive hydrocephalus. T1 sequences show hypointense abnormal signals (**Figure 3A**), and T2 sequences show hyperintense abnormal signals (**Figure 3B**). The “tiger-striped sign” could be found in the FLAIR sequence (**Figure 3C**), and scattered linear enhancement was seen after contrast (**Figure 3D**), with DWI sequence showing a slightly high signal (**Figure 3E**). The fourth ventricle and the aqueduct of Sylvius were compressed and narrowed, accompanied by adjacent brain tissue that was compressed and deformed (**Figure 3F**). The CBF pseudo-color map of the lesion shows high perfusion changes in red (**Figure 3G**). Single-voxel MRS examination shows the NAA/Cr ratio being 1.51 and the Cho/Cr ratio being 0.55 (**Figures 3H, I**). Because the lesion showed significant mass effect on the fourth ventricle with early signs of obstructive hydrocephalus, the patient underwent partial tumor resection and related treatment (**Figures 3J–P**), with good postoperative recovery. CT showed multiple irregular calcifications within the lesion, and all calcifications disappeared after surgery. (**Figures 3Q, R**). The final diagnosis was left cerebellar–brainstem–spinal LDD, CNS WHO Grade I. The patient’s preoperative KPS score was 70, as she could care for herself but was unable to carry on normal activities due to cerebellar dysfunction and intermittent headache. Following subtotal resection, her 1-month postoperative KPS score remained at 70 with minimal improvement in cerebellar signs. At 3-month follow-up, her score improved to 80, and by 6 months postoperatively, she maintained a KPS score of 80, with persistent but stable mild cerebellar dysfunction that required some effort during normal activities. The total follow-up period was 24 months (telephone-based), and the latest postoperative MRI was performed at month 6 (**Figures 3J–O**).

## Discussion

LDD is an extremely rare benign dysplastic tumor-like lesion, usually located in the cerebellar hemisphere and grows slowly (17). A significant milestone of LDD was the discovery of the connection between LDD and CS (6). CS is characterized by skin and mucosal rashes, intestinal polyps, and malignant lesions in the thyroid, breast, urinary reproductive system, and endometrium (18). It is an autosomal dominant inherited cancer syndrome, often accompanied by megalocephaly, gray matter abnormalities, polydactyly, meningocele, angiomyolipoma, macrocephaly, and macroglossia (19–24). It was further pointed out that LDD often coexists with “other” pathologies rather than as an isolated case, suggesting that LDD is a component of CS (13). Common clinical manifestations related to the mass effect of the cerebellum and ventricles include headaches, hydrocephalus, visual impairments, and cerebellar signs and symptoms (13, 14, 25).

### Epidemiology

**Figure 4:**
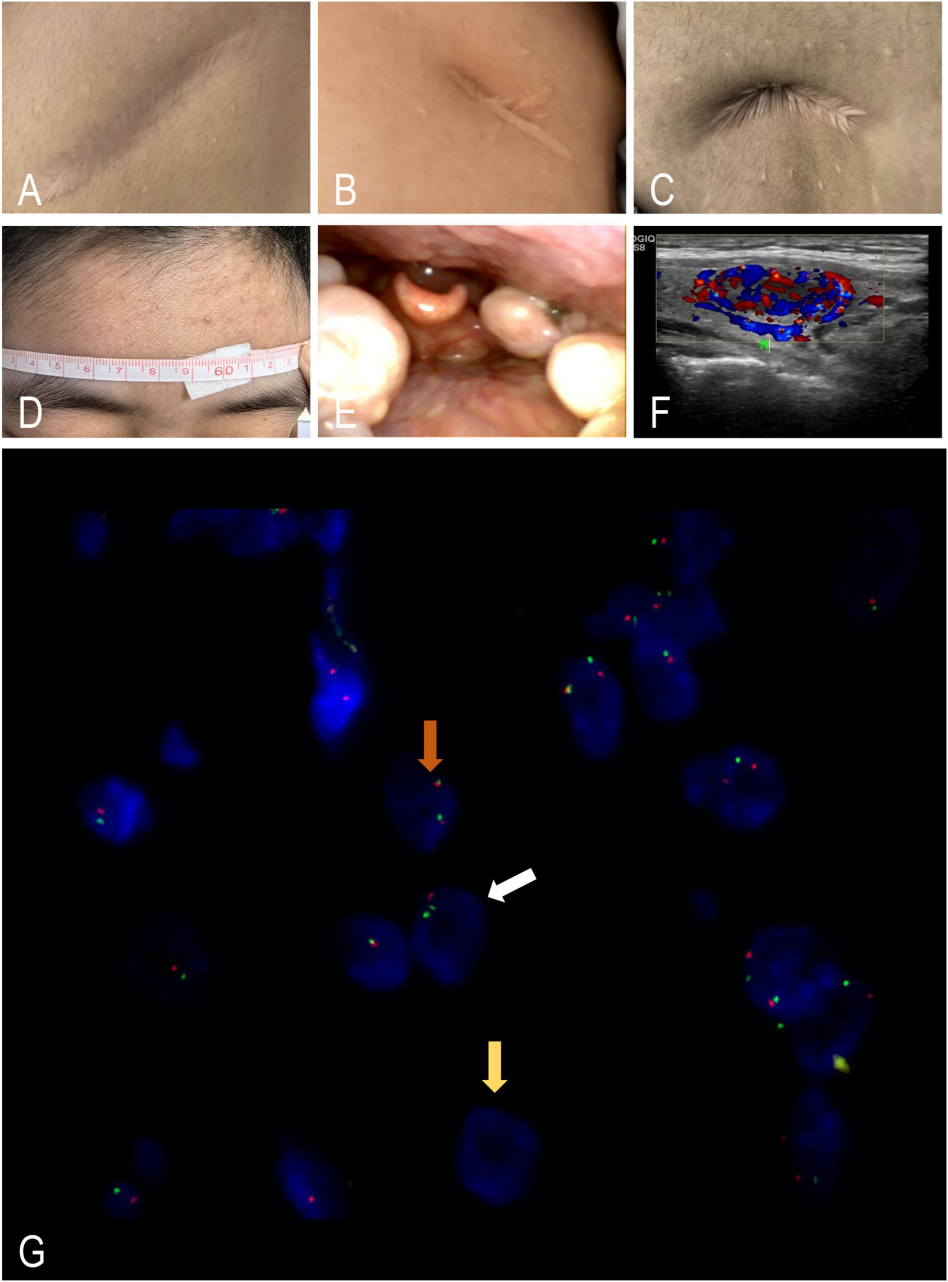
Evidence of Cowden syndrome in case 3. **(A–C)** Multiple subcutaneous cysts in the backside (lack of postoperative pathological examination). **(D)** Macrocephalus. **(E)** Thornwaldt cyst in the posterior parietal wall of the nasopharynx. **(F)** Multiple thyroid nodules. **(G)** 39% loss of PTEN heterozygosity and 5% PTEN homozygous deletion. PTEN (10q23) is labeled with orange–red signals, and the assessment is performed by counting signals in 100 tumor cells under fluorescence microscopy. Normal cell: brown arrows (2Red), PTEN heterozygosity: white arrows (1Red), PTEN homozygous deletion: yellow arrows (0Red).

LDD is rare, with only a few hundred cases reported worldwide across decades. It predominantly affects young to middle-aged adults, though cases occur across the lifespan, and a mild female predominance has been variably observed. As of September 2023, over 300 cases have been reported (1, 6–10, 17, 19, 22–24, 26, 27). It was proposed that LDD is a cranial manifestation of CS (1–12, 17–19, 26, 27). LDD is primarily an adult disease, which may explain the predominance of adult-onset CS (6). The onset age is mostly between 30 and 40 years old, with a higher incidence in women. Patients require genetic testing to rule out CS (28). In order to detect comorbid diseases of CS at the early stage, the NCCN guidelines have already included adult-onset LDD as one of the characteristic lesions of CS and recommend systemic physical testing and *PTEN* gene testing (13, 14, 29, 30). For patients with CS, the NCCN guidelines recommend monthly breast self-examinations (for women) after the age of 18, annual breast X-ray examinations for female patients after the age of 30–35, and annual thyroid ultrasound and full-body examinations (13, 14, 25). However, pediatric patients with LDD did not exhibit CS characteristics or *PTEN* mutations (31, 32), suggesting that adult-onset LDD should be considered a symptomatic standard of CS (33). Additionally, there were no significant differences in the incidence, recurrence, or mortality rates of CS between adult and pediatric patients according to reports (34). In this group of three adult patients, one 18-year-old female patient in this group showed obvious manifestations of CS, such as multiple subcutaneous cysts in the backside (**Figures 4A–C**), macrocephalus (**Figure 4D**) (the patient consented to publication of her image), Thornwaldt cyst in the nasopharynx (**Figure 4E**), and multiple thyroid nodules (**Figure 4F**). To confirm the diagnosis, fluorescence *in situ* hybridization (FISH) of *PTEN* was performed. The results show 39% loss of PTEN heterozygosity and 5% PTEN homozygous deletion (**Figure 4G**). Given its association with PTEN hamartoma tumor syndrome (PHTS), the management of LDD presents unique challenges in neurosurgical practice. This study aims to analyze the surgical outcomes of patients with LDD and evaluate the correlation between clinical presentation, genetic findings, and treatment outcomes, thereby establishing an evidence-based management strategy for this rare condition.

### MRI findings

In most cases of LDD, its unique imaging features are significant for preoperative diagnosis and determining the resection extent of lesions (35). Lesions are not enhanced or are slightly enhanced on contrast-enhanced scans (31). The abnormal proliferation of neuronal cells and excessive myelination in the cerebellar lobules show isointense signals on T1WI and T2WI, while the cerebrospinal fluid in the loose structure caused by central white matter atrophy shows a hypointense signal on T1WI and a hyperintense signal on T2WI, forming the typical MRI appearance of the disease, which is called “tiger stripe sign” (1, 23, 36, 37). The lack of enhancement in the lesion suggests no significant disruption of the blood–brain barrier (38). Building on these imaging observations, **Table 1** offers a concise comparison of LDD versus medulloblastoma, hemangioblastoma, and pilocytic astrocytoma by age predilection, typical location, MRI signal and enhancement patterns, diffusion/perfusion metrics, spectroscopy, syndromic links, and diagnostic histology (**Table 1**).

**Table 1:** Key features distinguishing Lhermitte–Duclos disease and common posterior fossa tumors.

Feature	Lhermitte–Duclos disease (LDD)	Medulloblastoma	Hemangioblastoma	Pilocytic astrocytoma (cerebellar)
Age predilection	Young to middle-aged adults (can occur across the lifespan)	Children/adolescents	Young–middle-aged adults; often von Hippel–Lindau (VHL) disease-associated	Children/adolescents
Typical location	Cerebellar hemisphere/vermian folia	Midline vermis	Cerebellar hemisphere; classically cyst with mural nodule	Cerebellar hemisphere; cyst–nodule pattern
T1/T2 signal	T1 low/iso; T2 high with laminated “tiger-stripe” striations	T1 low; T2 high (solid mass)	Cyst with T2-hyperintense cystic component; enhancing mural nodule	Cyst with T2-hyperintense cyst and enhancing nodule
Contrast enhancement	None or faint linear/striated	Avid, solid enhancement	Marked enhancement of mural nodule	Enhancing mural nodule
Diffusion (DWI)	Typically no restriction	Restriction common (low ADC)	Usually no restriction	Usually no restriction
Perfusion	Low to normal	High perfusion	High perfusion in the nodule	Moderate perfusion (variable)
MR spectroscopy (MRS)	NAA↓; choline not markedly elevated; ± lactate/lipid	Choline↑, NAA↓; ± taurine peak (in pediatrics)	Metabolically active mural nodule	Mild choline↑; NAA↓; ± lactate
Syndromic associations	Cowden syndrome / PTEN hamartoma tumor syndrome	None typical	von Hippel–Lindau (VHL) disease	Neurofibromatosis type 1 (NF1), rare
Key histopathology	Thickened molecular layer, Purkinje cell loss, hypertrophic ganglion-like neurons	Malignant small round blue cell tumor	Thin-walled capillaries with stromal cells	Pilocytic/biphasic astrocytoma with Rosenthal fibers and myxoid change

Demographic and clinical comparison between our three female patients (mean age: 38.3 years) and Kolhe’s six cases (66.7% female, mean age: 24.3 years) reveals similar female predominance but an older age distribution in our cohort (35). While headache was common in both series (66.7% vs. 83.3%), cerebellar signs and gait disturbances were more frequent in Kolhe’s patients (83.3% vs. 33.3%), potentially reflecting differences in lesion location or referral patterns. All our patients exhibited the pathognomonic “tiger-striped appearance” on T2-MRI compared to only 33.3% in Kolhe’s series, likely due to advanced imaging techniques and the greater recognition of this sign. Our study also incorporated multimodal imaging including MRS and perfusion studies not detailed in Kolhe’s report. Surgical approaches differed significantly, with only 33.3% of our patients undergoing gross total resection versus Kolhe’s apparent preference for radical excision despite acknowledging technical difficulties due to ill-defined tumor margins.

### Management

Surgical complete resection of the lesion, which is relatively good for prognosis (39), is the best method for this benign tumor (**Supplementary Figures 1A–F**). In principle, removing the tumor with the greatest extent possible while minimizing damage to brain tissue, relieving the lesion’s pressure on the surrounding brain tissues, and avoiding postoperative complications and recurrence as much as possible are the main goals of surgery (40). The residual lesions in most patients who undergo partial resection remain stable over the long term (5). By maintaining strict aseptic technique during surgery and preventing intracranial infection and postoperative cerebrospinal fluid (CSF) leakage, most patients can achieve favorable survival outcomes (1, 6–9, 12). The surgical intervention benefits include symptom resolution, prevention of progression, and definitive histological diagnosis. The potential risks include postoperative cerebellar edema and permanent neurological deficit.

The significant postoperative cerebellar edema in Case 1 might result primarily from two factors: manipulation of deep cerebellar white matter causing microvascular trauma and compromised venous outflow due to the tumor’s proximity to transverse and sigmoid sinuses. This complication highlights critical considerations for LDD resection. Prevention strategies should include the following: (1) preoperative venous architecture assessment with MRV, (2) maintaining a safety margin from major venous sinuses, and (3) considering subtotal resection when tumor tissue firmly adheres to venous structures. Given the LDD’s indolent nature, the risk–benefit ratio favors cautious surgical approaches that prioritize venous preservation over complete resection, especially in cases where the lesion interfaces with critical venous drainage pathways.

The role of radiotherapy in LDD management remains controversial and relatively unexplored compared to surgical options. Conventional radiotherapy has historically been reported as ineffective for LDD (35, 38). However, emerging evidence suggests that stereotactic radiotherapy (SRT) or stereotactic radiosurgery (SRS) may have a role in specific scenarios; when complete surgical excision is not achievable due to the involvement of critical neurovascular structures, adjuvant SRT may be considered to control the residual disease (41). For patients with recurrent LDD after a previous surgery who are poor surgical candidates or refuse reoperation, elderly patients, or those with significant comorbidities, SRT offers a non-invasive alternative (42).

For asymptomatic or mildly symptomatic patients, observation with structured MRI surveillance is reasonable. In surgically challenging or recurrent cases, SRT may be considered on a case-by-case basis. Given the PTEN–mechanistic target of rapamycin (mTOR) pathway involvement, mTOR inhibitors have shown anecdotal benefit in isolated reports; however, data remain limited and such therapy should be individualized.

### Histopathology

Microscopically, the characteristics of LDD were described as follows: the molecular layer is thickened, the Purkinje cell layer is markedly attenuated or absent, and the granular layer is replaced by hypertrophic ganglion-like neurons with enlarged nuclei and abundant cytoplasm, often with parallel myelination changes along folial architecture. The central white matter may show varying degrees of thinning; moreover, cerebellar cortices are hyperplastic, and the cerebellar gyri are widened (1). In our cases, we found that the molecular layer is thickened, the Purkinje cell layer is reduced or missing, and the granular cell layer contains a large number of abnormal neurons (**Supplementary Figures 1G–M**).

### Pathophysiology

Loss of PTEN function results in unchecked PI3K/AKT/mTOR signaling, promoting cellular hypertrophy, abnormal dendritic arborization, and altered myelination within cerebellar folia. These changes underlie the characteristic laminar thickening and “tiger stripe” appearance on MRI. Immunohistochemistry and prior series have demonstrated pathway activation in LDD specimens, supporting a hamartomatous process driven by PTEN dysregulation (43). The understanding of LDD’s molecular pathogenesis, particularly its association with germline PTEN mutations and dysregulation of the PI3K/AKT/mTOR pathway, has opened avenues for targeted pharmacological interventions. Rapamycin (sirolimus) and its analogs (rapalogs), which inhibit mTOR signaling, have shown promise in case reports (44). Zak et al. reported significant clinical improvement in an infant with bilateral cerebellar LDD following rapamycin initiation (45). Similarly, mTOR inhibitors like everolimus may have theoretical benefit, particularly in patients with concomitant CS manifestations. These approaches are especially valuable in patients unsuitable for surgery or radiotherapy, adjunctive therapy for residual or progressive disease, and management of multifocal disease in CS. Given the association with CS/PHTS, patients require long-term surveillance for systemic malignancies, including breast, thyroid, and endometrial cancers, according to contemporary guidelines. Genetic counseling and family cascade testing should be discussed.

It must be emphasized that the evidence for non-surgical management options remains limited due to the rarity of LDD. Treatment decisions should be made on a case-by-case basis with careful consideration of risk–benefit ratios and thorough discussion with patients regarding the advantages and limitations of each approach.

### Limitations

This single-center retrospective series with a small sample size limits generalizability. Follow-up duration and availability of long-term postoperative MRI varied among cases, and not all patients underwent comprehensive genetic testing or non-surgical interventions. Given the limited sample size, the conclusions should be interpreted with caution; these findings primarily aim to inform clinical recognition and surveillance and require confirmation in larger cohorts.

## Conclusion

Based on our experience and literature review, we recommend integrating PTEN assessment into the diagnostic workup of adult LDD, tailoring surgical extent to symptoms, mass effect, and venous anatomy, and adopting structured MRI surveillance (3–6 months postoperatively, then annually; more frequent for residual disease). Given the association with CS, clinicians should initiate genetic counseling and adhere to malignancy screening recommendations. Future multicenter studies are needed to refine non-surgical strategies and long-term outcomes.

## Data Availability

The raw data supporting the conclusions of this article will be made available by the authors, without undue reservation.

## Supplementary material

Supplementary Figure 1 Representative images of the procedure and pathological diagnosis are from the same patient during surgery. **(A)** The cerebellar hemisphere appears swollen, resembling fish flesh, showing a pale or light red color, with unclear boundaries from normal brain tissue. **(B)** The cerebellar gyri are widened, the cortex is thickened, and the structure is arranged in layers, similar to the layers of an onion. **(C)** The tumor and the enlarged cerebellar gyri resemble brain sulci, with blood vessels distributed among them. **(D)** The tumor protrudes downward towards the foramen magnum of the occipital bone, with no boundary from the brain tissue. **(E)** The tumor appears enlarged like brain gyri, with a rich blood supply distributed within the brain sulci. **(F)** Postoperative fluorescence staining of the tumor showed no obvious staining, indicating that the tumor was completely removed. **(G, H)** In case 1, mildly heteromorphic ganglionic cells with large nuclei and abundant cytoplasm were found, accompanied by mild hyperplasia of glial cells. The cerebellar gyrus, granular cells, and Purkinje cell layers were not clearly seen. **(I, J)** The cortical gyri in tumor tissue were thickened and poorly stratified. **(K)** The granular layer and Purkinje cell layer disappeared, accompanied by the proliferation of anomalous ganglion cells with large nuclei. Peripheral glial cell proliferation, mild cell atypia, and myelination changes parallel to the gyrus were observed inside the tumor. **(L, M)** The tumor tissue was swollen and disorganized in stratification, and gangliocytoid tumor cells with large nuclei were observed in all layers. Neoplastic ganglion cells were widely distributed in the molecular layer, and the surrounding glial cells were mildly hyperplastic. No necrosis or microvascular hyperplasia was observed.
