# Supplementary figures and images for: Case Report: A case series of Lhermitte–Duclos disease with surgical intervention
Source: Front Oncol. 2025 Oct 23;15:1552495. doi: 10.3389/fonc.2025.1552495 (PMC12588853; doi:10.3389/fonc.2025.1552495)

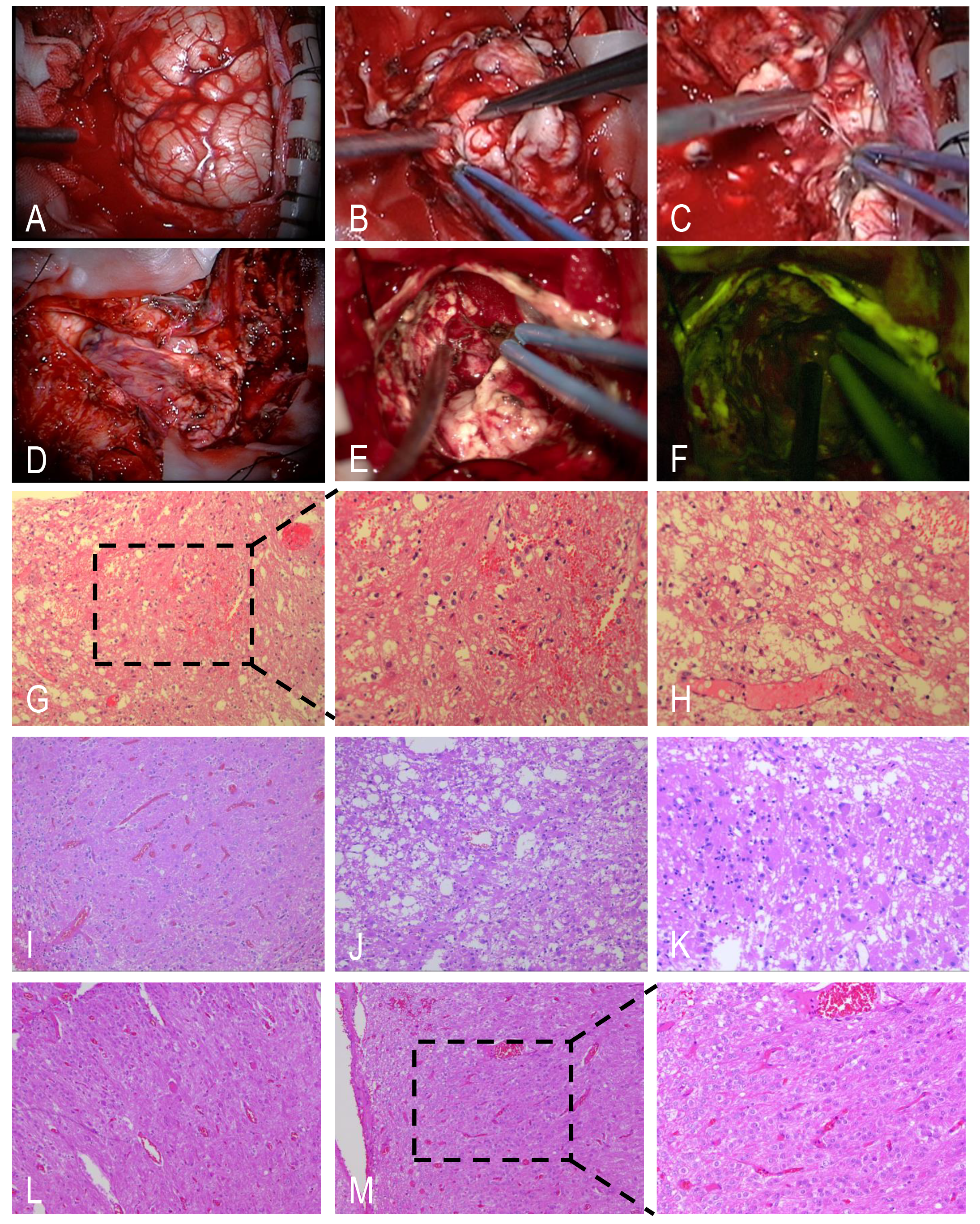

Supplement: Supplementary Figure 1 — Representative images of the procedure and pathological diagnosis are from the same patient during surgery. (A) The cerebellar hemisphere appears swollen, resembling fish flesh, showing a pale or light red color, with unclear boundaries from normal brain tissue. (B) The cerebellar gyri are widened, the cortex is thickened, and the structure is arranged in layers, similar to the layers of an onion. (C) The tumor and the enlarged cerebellar gyri resemble brain sulci, with blood vessels distributed among them. (D) The tumor protrudes downward towards the foramen magnum of the occipital bone, with no boundary from the brain tissue. (E) The tumor appears enlarged like brain gyri, with a rich blood supply distributed within the brain sulci. (F) Postoperative fluorescence staining of the tumor showed no obvious staining, indicating that the tumor was completely removed. (G, H) In case 1, mildly heteromorphic ganglionic cells with large nuclei and abundant cytoplasm were found, accompanied by mild hyperplasia of glial cells. The cerebellar gyrus, granular cells, and Purkinje cell layers were not clearly seen. (I, J) The cortical gyri in tumor tissue were thickened and poorly stratified. (K) The granular layer and Purkinje cell layer disappeared, accompanied by the proliferation of anomalous ganglion cells with large nuclei. Peripheral glial cell proliferation, mild cell atypia, and myelination changes parallel to the gyrus were observed inside the tumor. (L, M) The tumor tissue was swollen and disorganized in stratification, and gangliocytoid tumor cells with large nuclei were observed in all layers. Neoplastic ganglion cells were widely distributed in the molecular layer, and the surrounding glial cells were mildly hyperplastic. No necrosis or microvascular hyperplasia was observed. [file Image1.tif]
